# Lipoid proteinosis in a 36-year-old woman: An index case from Qatar

**DOI:** 10.5339/qmj.2026.43

**Published:** 2026-06-24

**Authors:** Engy Abdelhamed, Shajitha Thekke Veettil

**Affiliations:** 1 Directorate of Operations, Airport Health Centre, Primary Health Care Corporation, Doha, Qatar; 2 Directorate of Clinical Affairs, Department of Clinical Research, Primary Health Care Corporation, Doha, Qatar

**Keywords:** Lipoid proteinosis, extracellular matrix protein 1, blepharosis, temporal lobe, larynx, Qatar

## Abstract

**Background:**

Lipoid proteinosis (LP) is a rare autosomal recessive disorder caused by mutations in the extracellular matrix protein 1 (*ECM1*) gene. Reporting index cases is important for enhancing clinical recognition and understanding phenotypic variability.

**Case presentation:**

A 36-year-old woman presented with bilateral pruritic eyelid papules and long-standing hoarseness. Clinical examination revealed moniliform blepharosis, oral cobblestone plaques, and a broad tongue. Neuroimaging showed symmetrical calcifications in the medial temporal lobes. Fiberoptic laryngoscopy demonstrated an infantile larynx, an uncommon finding in LP. A positive family history and parental consanguinity supported the diagnosis. The patient declined genetic testing and neurological treatment despite experiencing a seizure episode and mood disturbances. Topical and systemic corticosteroids were ineffective. She continues ophthalmology follow-up but has discontinued dermatological and genetic care.

**Conclusion:**

This case highlights the classic features of LP, a potentially novel laryngeal finding, and emphasizes the need for early diagnosis and multidisciplinary management to address neurological and psychosocial complications.

## 1. INTRODUCTION

Lipoid proteinosis (LP), also known as Urbach–Wiethe disease or *hyalinosis cutis et mucosae*, is an exceedingly rare autosomal recessive disorder caused by mutations in the extracellular matrix protein 1 (*ECM1*) gene. This results in the deposition of periodic acid-Schiff (PAS)-positive, hyaline-like material in the skin, mucous membranes, brain, and other organs, affecting males and females equally.^1^ Although LP has been reported worldwide, fewer than 400 cases have been documented in the medical literature.^2–4^ Approximately 25% of reported cases originate from South Africa, particularly among individuals of Dutch or German ancestry.^5^

LP is characterized by a wide spectrum of cutaneous and mucosal manifestations, along with neurological, psychiatric, and gastrointestinal involvement.^6–9^ Disease onset typically occurs in early infancy and often presents with a weak cry or hoarseness due to laryngeal infiltration.^10^ In severe cases, progressive laryngeal involvement may lead to airway compromise. Over time, patients may develop diffuse skin thickening, papular lesions, and varioliform scarring. Infiltration of the tongue and frenulum can restrict tongue mobility, resulting in speech difficulties. Intracranial calcifications—most commonly involving the temporal lobes or hippocampus—have been reported and may be associated with seizures and neuropsychiatric complications.^11,12^

Despite its chronic course, individuals with LP generally have a normal life expectancy.^3^ However, neurological and psychiatric manifestations may significantly impair psychosocial functioning and quality of life.^13,14^

A systematic literature search was conducted to identify previously reported cases of LP from Qatar. PubMed, Embase, and Scopus were searched using combinations of the terms “lipoid proteinosis,” “Urbach–Wiethe disease,” and “Qatar.” No published case reports or case series from Qatar were identified. Although this does not exclude the existence of unreported cases, it suggests a lack of documented clinical experience with LP in the country. The absence of local data may contribute to limited awareness and delayed diagnosis. This case is presented to enhance clinical recognition and encourage further reporting of this rare disorder in the region.

## 2. CASE PRESENTATION

**Figure 1.**
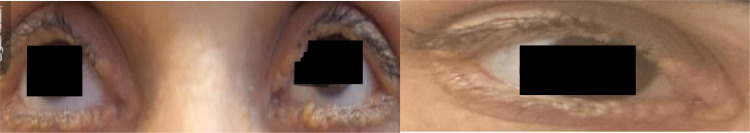
Photographs of eyelid lesions.

A 36-year-old woman presented to a family medicine clinic with three-year persistent, pruritic lesions affecting both eyelids (Figure 1), previously associated with intermittent pain and visual disturbances. She also reported longstanding cutaneous lesions over the upper limbs, thighs, and nasal bridge since childhood, along with a weak cry at birth and progressive hoarseness noted in early childhood.

**Figure 2.**
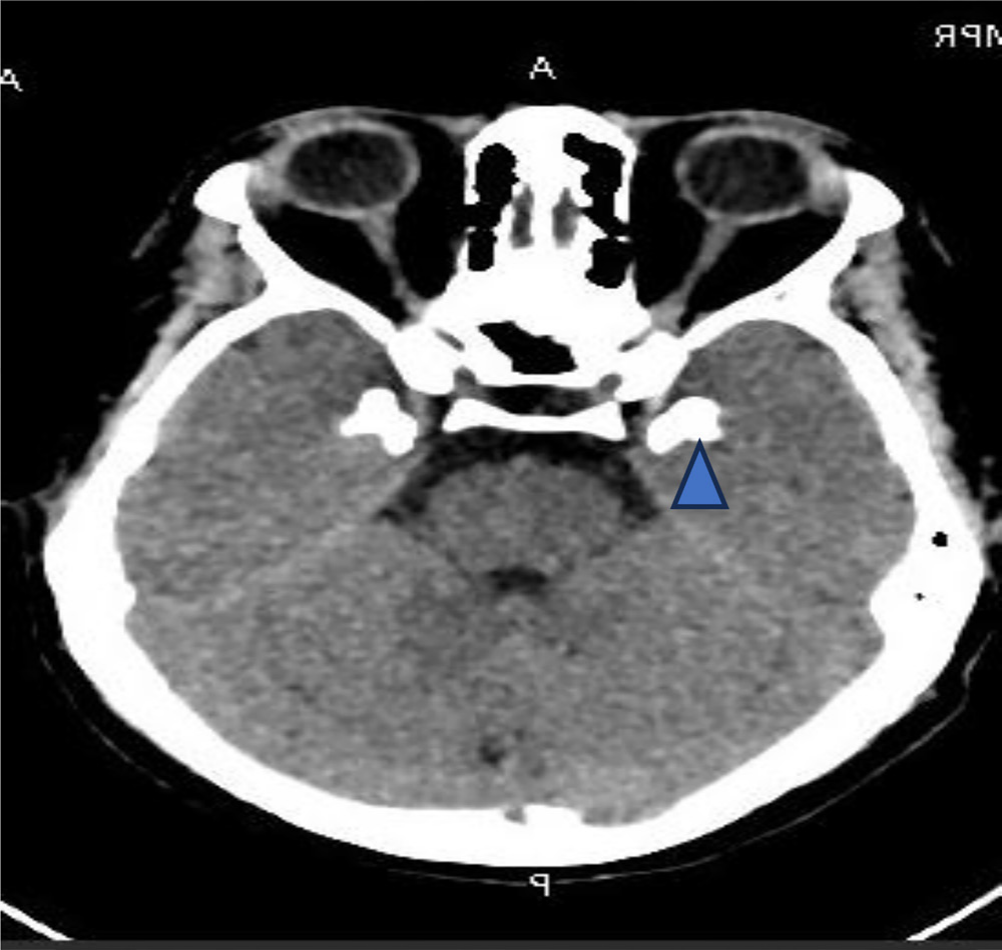
CT scan of the head showing the bilateral symmetrical dense calcifications.

She was diagnosed with LP based on magnetic resonance imaging (MRI) findings. Approximately two years prior, she experienced a seizure episode, following which a non-contrast computed tomography (CT) scan of the head demonstrated bilateral, symmetrical dense calcifications involving the medial temporal amygdala, consistent with LP (Figure 2). These findings represented a redemonstration of abnormalities previously noted on CT imaging performed 11 years earlier, which showed similar bilateral medial temporal amygdala calcifications, correlating with earlier MRI findings and the established clinical diagnosis. Her family history was significant for parental consanguinity and a similarly affected sibling.

**Figure 3.**
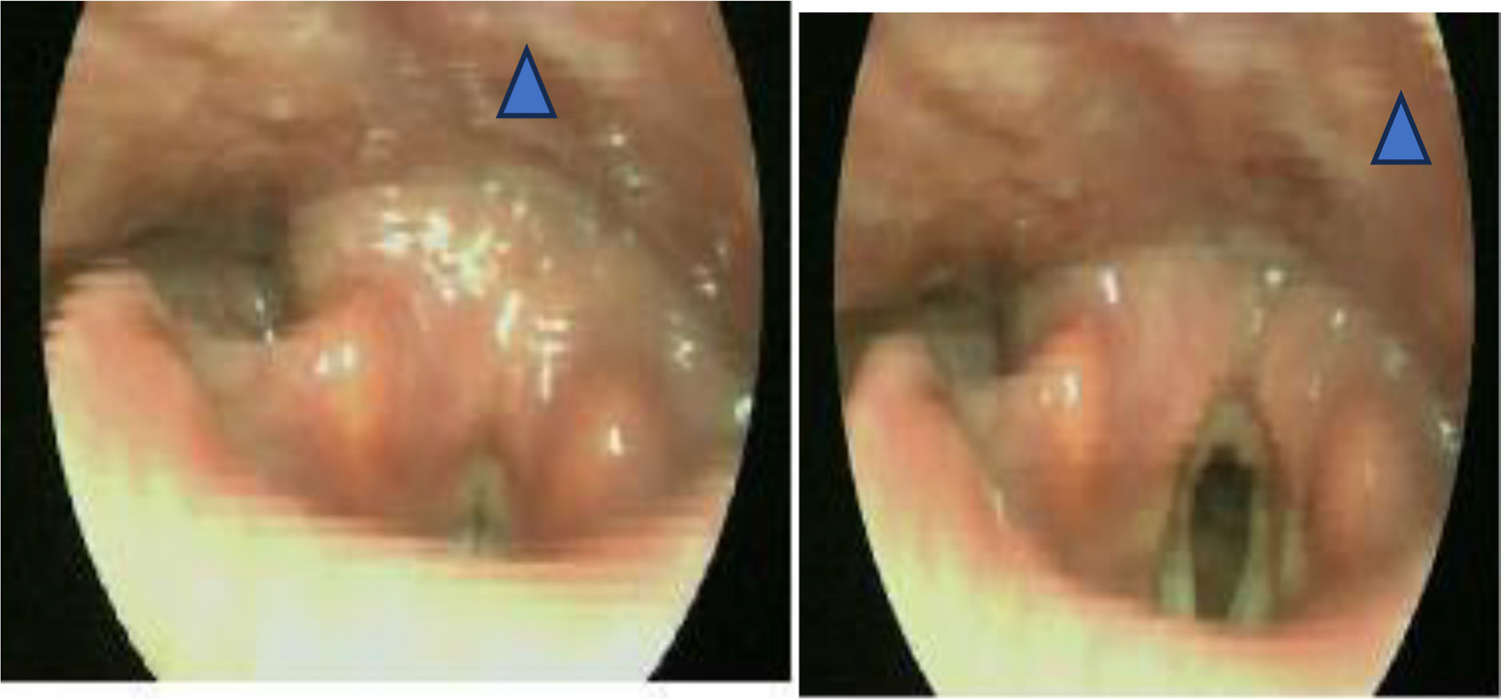
Fiberoptic laryngoscopy showing laryngeal infiltration.

Physical examination revealed beaded, waxy papules along the eyelid margins consistent with moniliform blepharosis. Cobblestone plaques were observed on the oral mucosa, along with infiltrated plaques over the arms. Fiberoptic laryngoscopy demonstrated an infantile larynx (Figure 3), characterized by an underdeveloped, small, high-set, child-like laryngeal configuration, with thickened, stiff vocal cords, narrowing of the laryngeal lumen, and mucosal infiltrates.

The patient was treated with topical and systemic corticosteroids without significant clinical improvement. She continues follow-up with ophthalmology services with stable symptoms, but has discontinued dermatology, genetics, and neurology consultations.

## 3. PATIENT CONSENT AND ETHICAL APPROVAL

Written informed consent was obtained from the patient for the publication of this case report, including accompanying clinical details and images. The patient’s identity has been fully protected. Ethical approval for this case was granted by the Primary Health Care Corporation (PHCC) Department of Clinical Research Institutional Review Board under reference number BUHOOTH-D-25-00069. All ethical guidelines and institutional protocols were strictly followed throughout the documentation and reporting of this case. This study was conducted in accordance with the Declaration of Helsinki (1964).

## 4. DISCUSSION

Lipoid proteinosis (LP) is a rare autosomal recessive disorder characterized by progressive deposition of hyaline material within the skin, mucosa, and central nervous system. The hallmark clinical features include hoarseness of voice from early infancy, vesiculobullous and hemorrhagic lesions of the skin and oral mucosa, verrucous or keratotic plaques on extensor surfaces, and moniliform blepharosis, which is widely regarded as pathognomonic.^1^

In the present case, the patient exhibited a weak cry from birth, followed by progressive hoarseness in early childhood—features consistent with early laryngeal involvement in LP. Fiberoptic laryngoscopy demonstrated an infantile larynx characterized by an underdeveloped, high-set, child-like configuration with thickened, stiff vocal cords, narrowing of the laryngeal lumen, and mucosal infiltrates. This finding is not commonly reported in existing literature and may represent an underrecognized or novel manifestation of LP. The absence of confirmatory genetic testing for ECM1 mutations is a limitation of this report; however, the diagnosis was strongly supported by classical clinical features and characteristic neuroimaging findings.

A recent systematic review and meta-analysis published in May 2024, encompassing 154 LP cases, reaffirmed hoarseness as the most consistent and earliest clinical manifestation, although it is frequently overlooked or misattributed.^15–17^ Moniliform blepharosis remains a distinctive and diagnostically valuable sign, often facilitating recognition of the disease.^18^ The present case reinforces the importance of these early clinical cues, particularly in adult patients presenting with long-standing multisystem involvement.

Neurological manifestations are well documented in LP. Symmetric bilateral calcifications of the amygdala nuclei—often described as “horn-bean” shaped lesions in the mesial temporal lobes—are considered characteristic, particularly in long-standing diseases.^19,20^ In this patient, such calcifications were evident on both MRI (2012) and CT imaging (2022). The occurrence of a seizure episode and reported mood disturbances are consistent with known neuropsychiatric sequelae of LP.

From a pathophysiological perspective, ECM1 plays a regulatory role in extracellular matrix integrity and is thought to inhibit matrix metalloproteinase-9 (MMP-9), a protein highly expressed in the brain. Loss of ECM1 function may lead to altered matrix homeostasis, facilitating epileptogenesis and contributing to psychiatric manifestations.^21^ Consequently, individuals with LP frequently exhibit epilepsy, mood disorders, anxiety, psychosis, impaired social behavior, and deficits in emotional regulation.^22^ In this case, the patient’s social withdrawal, emotional dysregulation, and occupational difficulties further illustrate the psychosocial burden of the disease.

The clinical variability of LP necessitates consideration of a broad range of differential diagnoses involving cutaneous, laryngeal, neurological, and systemic manifestations.^15^ Careful clinical correlation and imaging are essential to distinguish LP from other conditions with similar presentations.

Therapeutic options for LP remain limited and largely symptomatic. In this case, topical and systemic corticosteroids failed to produce clinical improvement. Emerging evidence from recent systematic reviews suggests that low-dose oral acitretin may provide symptomatic benefit in selected patients, with a comparatively favorable safety profile.^17^ Despite treatment challenges, individuals with LP typically have a normal life expectancy.^3^ However, neurological and psychiatric involvement can substantially impair psychosocial functioning and overall quality of life.^13^

## 5. CONCLUSION

This case illustrates the classic clinical features of LP, including early-onset hoarseness and moniliform blepharosis, along with characteristic temporal lobe calcifications. The identification of an infantile larynx represents a potentially novel or underreported manifestation of the disease. The presence of neurological and psychosocial complications highlights the complex disease burden associated with LP. As the first documented case from Qatar, this report contributes valuable regional data and expands the existing clinical spectrum of LP.

## AUTHORS’ CONTRIBUTIONS

Both EA and STV contributed to the conception of the work and drafted and critically revised the manuscript for content and final approval. The authors take responsibility for all aspects of reliability and absence of bias in the data presented, as well as its interpretation.

## FUNDING

This study received no external funding.

## COMPETING INTERESTS

The authors have no conflicts of interest to declare.

## DISCLOSURE OF AI USE

No artificial intelligence (AI) tools or automated systems were used in the preparation or writing of this manuscript.

## ACKNOWLEDGEMENT

We would like to acknowledge the Qatar National Library for providing funding for the publication of this study.
